# Evaluation of an automated thresholding algorithm for the quantification of paraspinal muscle composition from MRI images

**DOI:** 10.1186/s12938-017-0350-y

**Published:** 2017-05-22

**Authors:** Maryse Fortin, Mona Omidyeganeh, Michele Crites Battié, Omair Ahmad, Hassan Rivaz

**Affiliations:** 1 PERFORM Centre, Concordia University, 7200 Sherbrooke W, Montreal, QC H4B 1R6 Canada; 2 Department of Electrical Engineering, Engineering, Computer Science and Visual Arts Integrated Complex, Concordia University, 1515 Ste-Catherine W. Street, Montreal, QC H3G 2W1 Canada; 3 Common Spinal Disorders Research Group, Faculty of Rehabilitation Medicine University of Alberta, 8205-114 Street, Edmonton, AB T6G 2G4 Canada

**Keywords:** Multifidus, Erector spinae, Paraspinal muscle, Fatty infiltration, Magnetic resonance imaging, Automated algorithm

## Abstract

**Background:**

The imaging assessment of paraspinal muscle morphology and fatty infiltration has gained considerable attention in the past decades, with reports suggesting an association between muscle degenerative changes and low back pain (LBP). To date, qualitative and quantitative approaches have been used to assess paraspinal muscle composition. Though highly reliable, manual thresholding techniques are time consuming and not always feasible in a clinical setting. The tedious and rater-dependent nature of such manual thresholding techniques provides the impetus for the development of automated or semi-automated segmentation methods. The purpose of the present study was to develop and evaluate an automated thresholding algorithm for the assessment of paraspinal muscle composition. The reliability and validity of the muscle measurements using the new automated thresholding algorithm were investigated through repeated measurements and comparison with measurements from an established, highly reliable manual thresholding technique.

**Methods:**

Magnetic resonance images of 30 patients with LBP were randomly selected cohort of patients participating in a project on commonly diagnosed lumbar pathologies in patients attending spine surgeon clinics. A series of T2-weighted MR images were used to train the algorithm; preprocessing techniques including adaptive histogram equalization method image adjustment scheme were used to enhance the quality and contrast of the images. All muscle measurements were repeated twice using a manual thresholding technique and the novel automated thresholding algorithm, from axial T2-weigthed images, at least 5 days apart. The rater was blinded to all earlier measurements. Inter-method agreement and intra-rater reliability for each measurement method were assessed. The study did not received external funding and the authors have no disclosures.

**Results:**

There was excellent agreement between the two methods with inter-method reliability coefficients (intraclass correlation coefficients) varying from 0.79 to 0.99. Bland and Altman plots further confirmed the agreement between the two methods. Intra-rater reliability and standard error of measurements were comparable between methods, with reliability coefficient varying between 0.95 and 0.99 for the manual thresholding and 0.97–0.99 for the automated algorithm.

**Conclusion:**

The proposed automated thresholding algorithm to assess paraspinal muscle size and composition measurements was highly reliable, with excellent agreement with the reference manual thresholding method.

## Background

Imaging assessment of paraspinal muscle morphology and fatty infiltration has attracted considerable attention over recent decades, with reports suggesting an association between muscle degenerative changes (e.g. atrophy, asymmetry, fatty infiltration) and low back pain (LBP) [1–7]. However, there remain inconsistencies in the literature related, in part, to variations in imaging modalities used, such as magnetic resonance imaging (MRI), ultrasound and computed tomography (CT), and measurement protocols.

To date, qualitative and quantitative approaches have been used to assess paraspinal muscle composition (e.g. fatty infiltration). Qualitative approaches involve the use of visual grading schemes to assess the degree of paraspinal muscle fatty infiltration on MR images. Recently, the reliability of measurements of lumbar multifidus fatty infiltration, using the Goutallier classification system (GCS) (0–4 grading scale) [8], which was initially developed to assess fatty degeneration in rotator cuff injuries, was assessed. Although such studies have reported good intra-rater (ICC or kappa 0.71–0.93) [8–10] and inter-rater reliability (ICC or kappa 0.58–0.85) [8–10], these methods do not provide precise measurement and are not suitable to evaluate changes over time. On the other hand, quantitative MRI measurements of paraspinal muscle composition are achieved by segregating pixels within the selected muscle region of interest that are thought to represent fat, using a manual segmentation or threshold method. As the signal intensity of each pixel from an MR image can be assigned a grey scale value, various thresholding techniques using different software applications have been used to quantify paraspinal muscle composition. Though highly reliable [11, 12], manual thresholding techniques are time consuming and not always feasible in clinical and some research settings, and the use of proprietary image analysis software and insufficient descriptions of measurement protocols hinder replication of results by others. As homogeneous tissue may have varying signal intensities (e.g. intensity bias) between subjects and within the same subject on different scan slices due to MR field inhomogeneity [12], the threshold limit representing lean muscle tissue needs to be identified for each subject and scan slice, making segmentation a time-consuming and complex task. The tedious and rater-dependent nature of such manual thresholding techniques for paraspinal muscle composition assessment provides the impetus for the development of automated or semi-automated segmentation methods. Although, automated and sophisticated methods have been successfully implemented in MR image tissue segmentation of different anatomical structures including the brain, liver, heart [13–16], as well as the quantification of thigh muscle and adipose tissue [17], we are aware of only one recently developed semi-automated interactive tool for the assessment of paraspinal muscle composition [18]. The threshold values in the interactive segmentation technique, however, are based on visual inspection and, therefore, remain rater-dependent.

The purpose of the present study was to develop and evaluate an automated thresholding algorithm for the assessment of paraspinal muscle composition. The reliability and validity of the muscle measurements using the new automated thresholding algorithm were investigated through repeated measurements and comparison with measurements from an established, highly reliable manual thresholding technique [11].

## Methods

### Sample of lumbar MRI

A sample of 30 patients (11 female and 19 male) was randomly selected from a cohort of patients participating in Genodisc, a European research consortium project on commonly diagnosed lumbar pathologies in patients attending spine surgeon clinics. All patients included in this study received a diagnosis of disc herniation, spinal stenosis, spondylolisthesis, or nonspecific LBP. Patients were excluded if they were below 18 or over 60 years of age, had a contract agent allergy, had reduced renal function, were not able to undergo MRI acquisition, or had a tumor, infection, spinal fracture, rheumatoid arthritis or were pregnant. All participants completed a consent form acknowledging that their data will be used for research purposes.

The MRI protocol included a routine T2-weighted turbo spin echo sequence for both axial and sagittal images acquired with a Siemens Avanto 1.5T MRI system (Siemens AG, Erlangen, Germany) (axial T2 parameters included repetition time = 4000, echo time = 113 and slice thickness = 3 mm).

### Automated thresholding algorithm

**Fig. 1:**
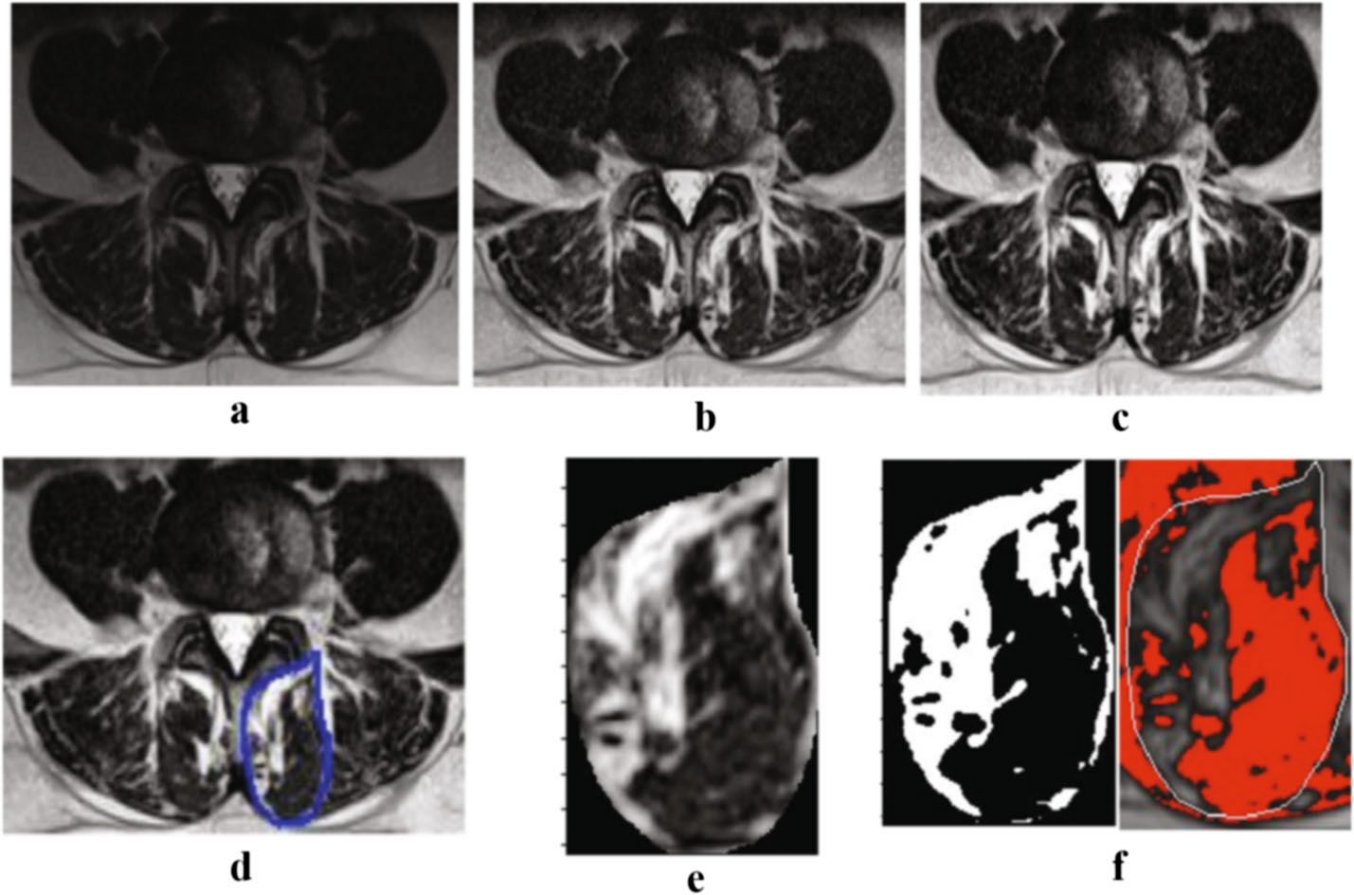
A sample MRI image at L4–L5 and the processed image after each step of the algorithm; **a** the original MRI image, **b** adaptive equalized algorithm image, **c** adjusted contrast image, **d** the select area, **e** the* cropped area* of the selected ROI, **f** the resulted binary image from automated algorithm (*left*) and manual thresholding technique (*right*)

Initially, a series of T2-weighted MR images from two patients were used to train the algorithm. Muscle measurements were then automatically calculated by the algorithm, which involves a series of steps, once the muscle of interest has been manually segmented. First, a preprocessing technique was applied to each MR image to enhance the quality and the contrast of the images. This preprocessing step includes an adaptive histogram equalization method and image adjustment scheme. The adaptive histogram equalization algorithm was employed to balance the grayscale level at each point of the image. We have used contrast limited adaptive histogram equalization (CLAHE) algorithm [19]. In this algorithm the histogram equalization is applied on small rectangles of the image instead of the whole image. It changes the histogram of each rectangle to a uniform distribution. A bilinear interpolation method was also applied to avoid the formation of artificially stimulated boundaries. Then, the image adjustment scheme was utilized to improve the contrast of the image. This modifies the contrast of the image so that only a small fraction (1%) of the image is saturated as low (dark) and high (bright) intensities [20, 21], providing a high contrast MR image (Fig. 1). These preprocessing steps were applied to reduce the inhomogeneity artifacts. Since our method increase the image contrast locally, the thresholding step was minimally affected by this noise.

In order to calculate the area of fat and muscle tissue, a threshold level was selected using the Otsus’s scheme [22, 23]. This threshold is calculated to minimize the interclass difference between black and white points, and normalized the pixel intensity values between 0 and 1. The chosen threshold value is then applied to the selected ROI, and the algorithm computes automatically the number of white and black pixels in the area, which will represent the area of fat and muscle tissue. As the MRI images used for this study were of high quality, the Otsu thresholding technique was adequate for our experiments. While the preprocessing steps to enhance the contrast of the image (as described above) provided a high contrast image and Otsu thresholding method segments the image with accuracy compatible with the manual segmentation. The algorithm was implemented in MATLAB (Mathworks, Natick, MA, USA).

### Muscle measurements

All muscle measurements were acquired by one of the investigators (MF), who has more than 6 years of experience in quantitative MRI muscle assessment. Quantitative measurements of the multifidus and erector spinae muscles were obtained from axial T2-weighted images at mid-disk for L4–L5 and L5–S1 for every subject. This image sequence was selected as it is routinely obtained in lumbosacral MRI examination and has been widely used to assess paraspinal muscle composition. The two levels were selected because most lumbar pathologies and muscle morphological changes occur at L4–L5 and L5–S1. The paraspinal muscle measurements of interest for this study included: the total cross-sectional area (CSA), the functional cross-sectional area (FCSA), representing the area of pure muscle mass (excluding fatty infiltration) and the area occupied by fat, and the fat percentage.

Muscle measurements were first obtained using a manual thresholding technique using ImageJ image analysis software (version 1.43, National Institutes of Health, Bethesda, Maryland). FCSA was measured by manually selecting a threshold signal within the total muscle CSA to include only pixels within lean muscle tissue range. The grayscale range for lean muscle mass was established for each subject and scan slice. This thresholding technique has been shown to be highly reliable and is described in detail elsewhere [11]. Once the first set of measurements with ImageJ was completed, the rater was blinded to the results and the same MRI slices were then assessed using the automated algorithm and MATLAB software (version R2015b), a minimum of 5 days after the first measurements were completed. For this method, the rater manually segmented the CSA of the muscle of interest on each slice, and the thresholding algorithm automatically calculated the muscle CSA, the fat CSA and the muscle fat percentage. All muscle measurements were obtained four times by the same rater, twice using the manual thresholding method and twice using the automated thresholding algorithm.

### Statistical analysis

Descriptive statistics, such as means and standard deviations, were calculated for each muscle measurement of interest. The ICC_(2,1)_ was calculated to determine the intra-rater reliability of measurement using the manual thresholding technique and automated algorithm, as well as the inter-method reliability using a two-way random-effects model and absolute agreement. The ICCs were interpreted using the following criteria, as suggested by Portney and Watkins: 0.00–0.49 = poor, 0.50–74 = moderate, and 0.75–1.0 = excellent [24]. Method agreement between the measurements acquired using the manual thresholding technique and the automated algorithm was also evaluated using the 95% limits of agreement, as suggested by Bland and Altman [25, 26]. The standard error of measurement (SEM) was calculated to provide an estimate of the expected error related to a particular measurement in the same units as the initial measurement (SEM = S√(1 − r_xx_), where S = standard deviation of the test, and r_xx_ = reliability of the test). Results were analyzed according to the spinal level and muscle investigated. The statistical analysis was performed using Statistical Package for the Social Sciences version 23.0 (SPSS Inc, Chicago, Illinois).

## Results

### Inter-method reliability of muscle measurements using the manual thresholding technique and automated algorithm

The results for the inter-method correlation (ICC), SEM values, and descriptive statistics (mean ± SD) for the right and left side of the multifidus and erector spinae are presented in Table 1. The inter-method reliability was analyzed by comparing the first set of measurements collected with each method. The ICCs for all of the different muscle composition measurements, regardless of the muscle analyzed, side or spinal level, showed excellent agreement and varied between 0.79 and 0.99. The SEM was also comparable for the different muscle measurements, muscle analyzed, side and spinal level.

**Table 1:** Inter-method reliability indexes between the manual thresholding technique and automated thresholding algorithm for the right and left multifidus and erector spinae muscles at L4–L5 and L5–S1

Parameter	Right side			Left side		
	Mean (SD)	ICC (95% CI)	SEM	Mean (SD)	ICC (95% CI)	SEM
Multifidus L4–L5						
CSA (cm^2^)	9.77 (1.83)	0.99 (0.98–1.00)	0.18	9.39 (1.44)	0.98 (0.97–0.99)	0.20
FCSA (cm^2^)	4.39 (1.58)	0.84 (0.68–0.92)	0.63	4.44 (1.55)	0.90 (0.76–0.95)	0.49
Fat CSA (cm^2^)	5.38 (1.38)	0.83 (0.65–0.92)	0.57	4.95 (1.10)	0.80 (0.51–0.91)	0.49
Fat %	0.56 (0.12)	0.79 (0.57–0.90)	0.05	0.53 (0.12)	0.83 (0.55–0.92)	0.05
Erector spinae L4–L5						
CSA (cm^2^)	16.36 (3.39)	0.98 (0.96–0.99)	0.48	17.35 (3.88)	0.99 (0.95–0.99)	0.39
FCSA (cm^2^)	7.25 (2.47)	0.96 (0.91–0.98)	0.49	9.64 (2.32)	0.94 (0.88–0.97)	0.57
Fat CSA (cm^2^)	9.11 (2.28)	0.94 (0.84–0.97)	0.56	9.64 (2.33)	0.91 (0.78–0.96)	0.70
Fat %	0.56 (0.10)	0.91 (0.81–0.95)	0.03	0.56 (0.10)	0.85 (0.70–0.93)	0.04
Multifidus L5–S1						
CSA (cm^2^)	11.25 (1.75)	0.97 (0.90–0.97)	0.30	11.29 (1.53)	0.98 (0.96–0.99)	0.22
FCSA (cm^2^)	5.34 (1.94)	0.93 (0.86–0.96)	0.51	5.49 (1.65)	0.90 (0.80–0.95)	0.52
Fat CSA (cm^2^)	5.91 (1.27)	0.86 (0.72–0.93)	0.48	5.80 (1.21)	0.78 (0.54–0.89)	0.57
Fat %	0.53 (0.13)	0.90 (0.80–0.95)	0.04	0.52 (0.11)	0.82 (0.62–0.91)	0.05
Erector spine L5–S1						
CSA (cm^2^)	11.26 (4.06)	0.97 (0.90–0.99)	0.70	11.43 (4.4)	0.95 (0.87–0.97)	0.98
FCSA (cm^2^)	3.77 (2.22)	0.91 (0.71–0.96)	0.67	3.88 (2.27)	0.94 (0.88–0.97)	0.62
Fat CSA (cm^2^)	7.49 (2.37)	0.97 (0.94–0.98)	0.41	7.55 (2.53)	0.91 (0.83–0.96)	0.68
Fat %	0.68 (0.11)	0.81 (0.48–0.92)	0.04	0.68 (0.10)	0.78 (0.50–0.90)	0.05

*ICC* intra-class correlation coefficient, *CI* confidence interval, *SEM* standard error of measurement, *CSA* cross-sectional area, *FCSA* functional cross-sectional area

### Inter-method agreement

**Fig. 2:**
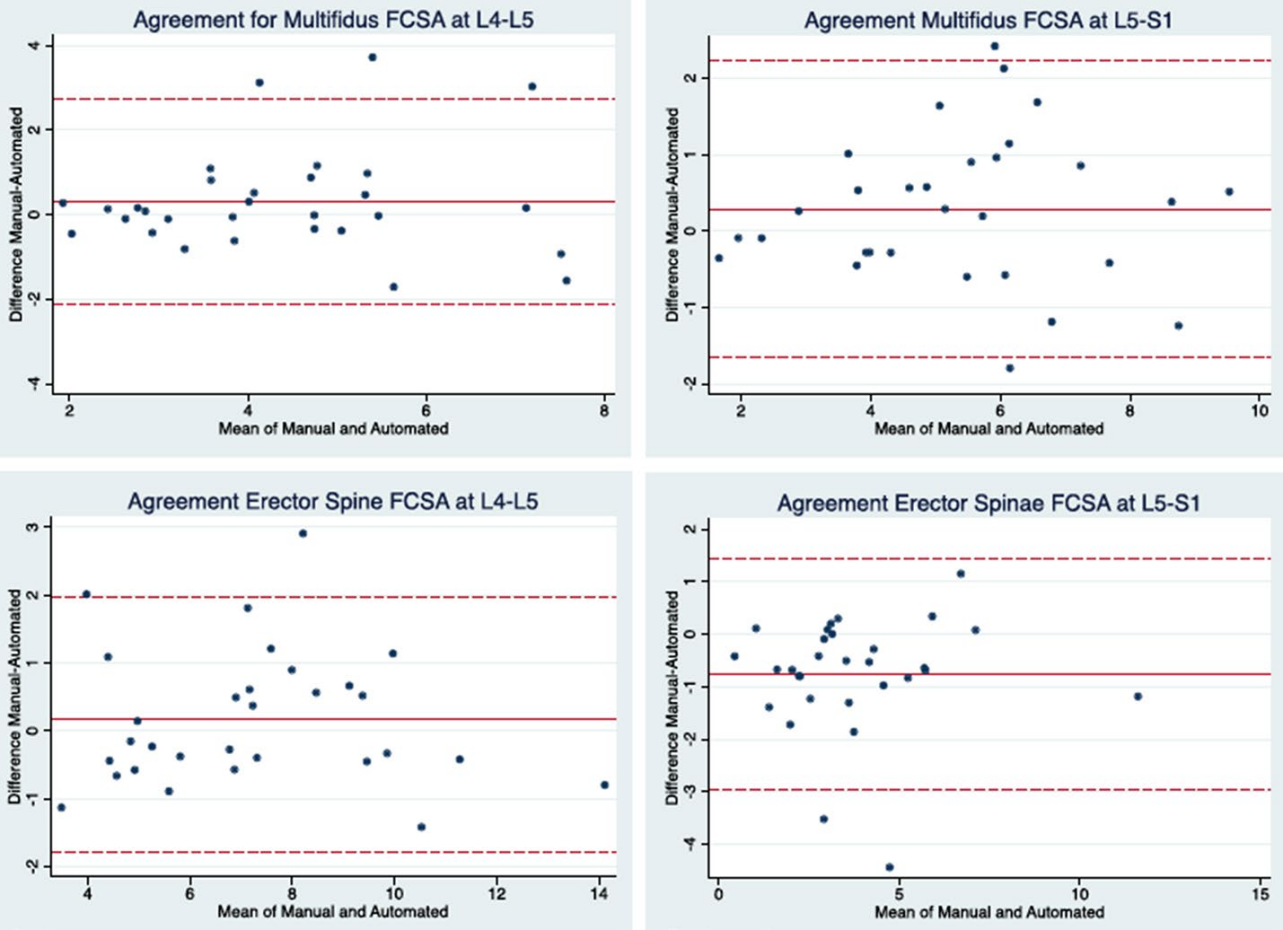
Bland–Altman 95% limits of agreement plots for the FCSA measurements of the multifidus and erector spinae muscles at L4–L5 and L5–S1

**Fig. 3:**
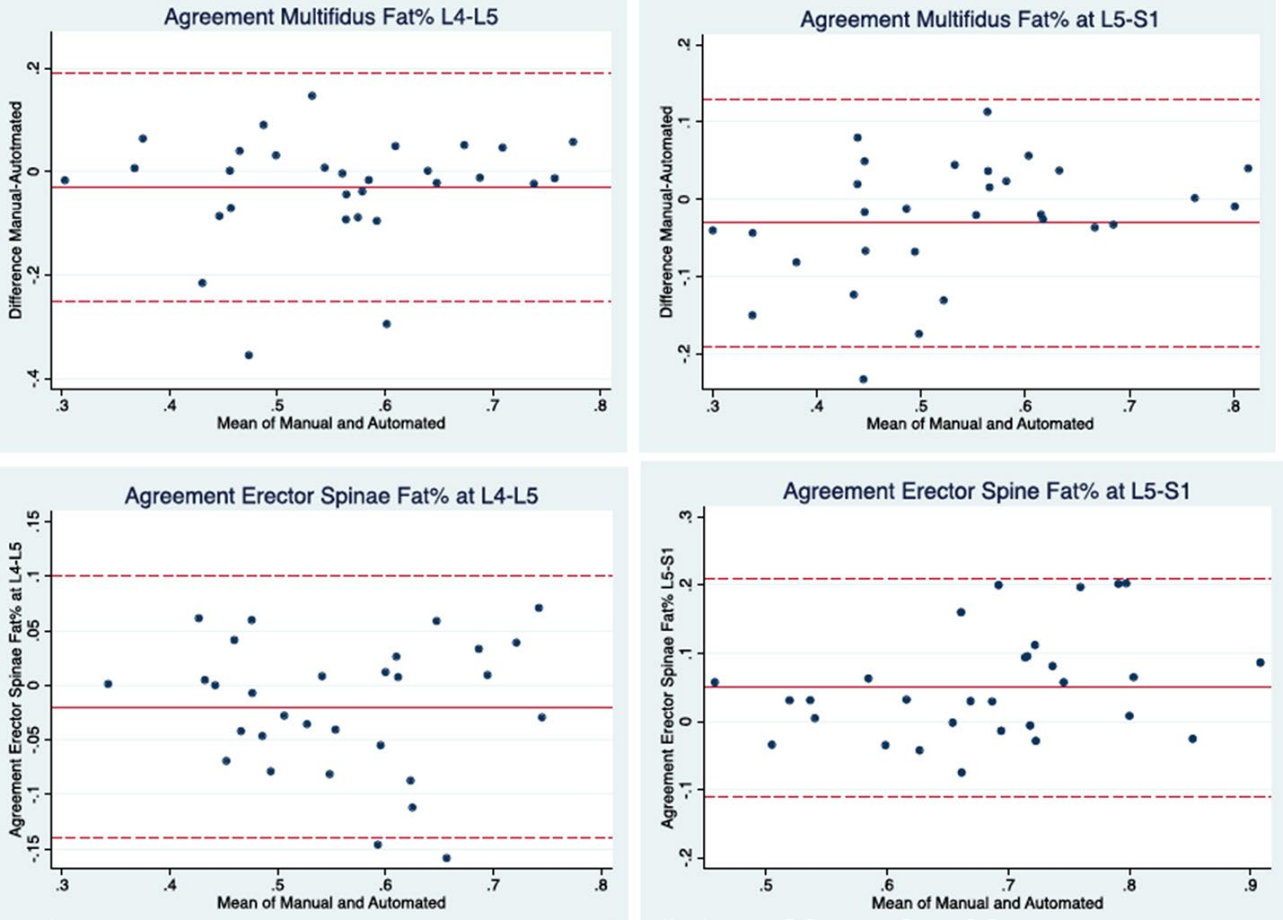
Bland–Altman 95% limits of agreement plots for the fat % measurements of the multifidus and erector spinae muscles at L4–L5 and L5–S1

Figures 2 and 3 show the combined Bland and Altman 95% limits of agreement plots for the FCSA and fat percentage measurement from the right multifidus and erector spinae at L4–L5 and L5–S1 using the first set of measurements collected using the manual thresholding technique and automated algorithm. Two methods are considered to have good agreement when the measurement difference is small enough for both methods to be used interchangeably [25]. In accordance with Bland and Altman, [26] all the plots show good agreement between the manual thresholding technique and automated algorithm and no systematic bias; the distribution of the scores around the mean approximates zero and is spread evenly and randomly above and below the line. A histogram of the difference scores was also prepared for every measurement parameter, and all histograms followed a normal distribution. As such, because the error is normally distributed, we can observe that about 95% of the points are between the limits of agreement (noted by the dashed lines on the plots) for each measure. The width of the limits of agreement is also small.

### Intra-rater reliability of muscle measurements using the manual thresholding technique and automated algorithm

The intrarater reliability (ICC), SEM values, and descriptive statistics (mean ± SD) related to the manual thresholding technique and automated algorithm for the right multifidus and erector spinae muscles at L4–L5 and L5–S1 are presented in Table 2. The results of the left side were virtually equivalent and are not presented. The ICCs for the intrarater reliability across both spinal levels for the manual thresholding technique ranged from 0.95 to 0.99 and 0.97 to 0.99 for the automated algorithm. The ICCs for the fat CSA and fat percentage measurements tended to be slightly lower for the manual thresholding technique, in comparison to the automated algorithm. The SEM associated with each muscle parameter was generally smaller for the measurements obtained with the automated algorithm as compared to the manual thresholding technique.

**Table 2:** Intra-rater reliability indexes for the manual thresholding technique and automated thresholding algorithm for the right multifidus and erector spinae muscles at L4–L5 and L5–S1

Parameter	Manual thresholding technique			Automated thresholding algorithm		
	Mean (SD)	ICC (95% CI)	SEM	Mean (SD)	ICC (95% CI)	SEM
Multifidus L4–L5						
CSA (cm^2^)	9.87 (1.81)	0.99 (0.97–1.00)	0.18	9.87 (1.85)	0.99 (0.97–1.00)	0.19
FCSA (cm^2^)	4.54 (1.79)	0.97 (0.93–0.99)	0.31	4.23 (1.66)	0.99 (0.99–1.00)	0.20
Fat CSA (cm^2^)	5.33 (1.42)	0.95 (0.89–0.97)	0.32	5.56 (1.39)	0.99 (0.97–1.00)	0.20
Fat %	0.55 (0.13)	0.95 (0.91–0.98)	0.03	0.57 (0.12)	0.99 (0.98–1.00)	0.01
Erector spinae L4–L5						
CSA (cm^2^)	16.31 (3.38)	0.99 (0.98–1.00)	0.34	16.67 (3.53)	0.98 (0.95–0.99)	0.49
FCSA (cm^2^)	7.25 (2.51)	0.98 (0.96–0.99)	0.35	7.18 (2.55)	0.97 (0.94–0.98)	0.44
Fat CSA (cm^2^)	9.06 (2.37)	0.96 (0.92–0.98)	0.34	9.49 (2.45)	0.99 (0.99–1.99)	0.25
Fat %	0.56 (0.11)	0.95 (0.89–0.97)	0.02	0.57 (0.11)	0.99 (0.99–1.00)	0.01
Multifidus L5–S1						
CSA (cm^2^)	11.40 (1.72)	0.99 (0.98–1.00)	0.17	11.34 (1.82)	0.98 (0.96–0.99)	0.26
FCSA (cm^2^)	5.34 (1.94)	0.97 (0.88–0.98)	0.33	5.26 (1.95)	0.99 (0.98–1.00)	0.19
Fat CSA (cm^2^)	6.06 (1.52)	0.95 (0.56–0.98)	0.34	6.08 (1.17)	0.99 (0.97–0.99)	0.12
Fat %	0.54 (0.14)	0.97 (0.78–0.99)	0.02	0.54 (0.12)	0.99 (0.98–1.00)	0.01
Erector spine L5–S1						
CSA (cm^2^)	11.03 (3.97)	0.99 (0.98–1.00)	0.40	11.65 (4.05)	0.98 (0.96–0.99)	0.57
FCSA (cm^2^)	3.24 (2.21)	0.98 (0.94–0.99)	0.31	4.09 (2.21)	0.97 (0.94–0.98)	0.38
Fat CSA (cm^2^)	7.78 (2.36)	0.97 (0.66–0.99)	0.41	7.56 (2.43)	0.98 (0.97–0.99)	0.34
Fat %	0.73 (0.12)	0.97 (0.62–0.99)	0.02	0.66 (0.10)	0.97 (0.94–0.98)	0.02

*ICC* intra-class correlation coefficient, *CI* confidence interval, *SEM* standard error of measurement, *CSA* cross-sectional area, *FCSA* functional cross-sectional area

## Discussion

We have presented a new automated thresholding algorithm for quantitative paraspinal muscle composition assessment based on MR images. The primary goal of this study was to examine to validity of the measurements obtained with the novel automated thresholding algorithm, as compared to those obtained with an established manual thresholding segmentation method. The correlation and agreement of the related paraspinal muscle measurements suggest that the two methods yield comparable measurements, with excellent reliability when applied to a clinically relevant population. These findings are further supported by the Bland and Altman limits of agreement that indicate inter-method agreement is within an acceptable range to use either of the two methods interchangeably. Moreover, the similar intra-rater reliability and SEMs indicate that the proposed automated algorithm produces results consistent with the reference manual thresholding method.

While paraspinal muscle composition (including the quantification measures of this study) have already been applied and investigated in different low back pain population, literature findings are controversial with regards to their predictive clinical value. Currently, a wide range of methodologies and modalities are used to assess paraspinal muscle composition, which is likely related to the inconsistent findings. The developed algorithm greatly simplifies the complexity and tedious aspect of MR imaging assessment of paraspinal muscle composition and provides a standardized procedure. More specifically, the results obtained using our novel automated thresholding algorithm are particularly encouraging and promising for the following reasons: (1) the threshold selection to identify the pixels representing muscle and fat tissue is completely automated, and thus easily reproducible, time efficient and rater-independent, while the manual thresholding method requires a trained rater to identify the threshold upper and lower limits, (2) the method is not affected by anatomical or image quality differences between subjects, (3) the automated algorithm can be readily used and applied to various datasets to produce robust measurements of paraspinal muscle composition. Furthermore, as could be expected with a largely automated system, the intra-rater reliability was slightly higher when the measurements were obtained with the automated thresholding algorithm, as compared to the manual method. Overall, the SEMs of the related paraspinal muscle composition parameters were also smaller when measurements were acquired with the automated algorithm. These findings reflect the higher precision of the algorithm in reproducing measurements. Furthermore, we suspect that spatial resolution of the MR images had a minimal impact on the accuracy of the segmentation. As the MR images used were selected from a database of patients that underwent a routine lumbosacral examination, the MRI parameters were very similar for each patients and the image quality and spatial resolution (e.g. pixel size between 1 and 2 mm) was representative of the images that would be used clinically. Lastly, although recent studies have demonstrated that MR imaging techniques such as fat-signal fraction using Dixon and multi-echo imaging (mostly in liver) may be superior in quantifying aqueous tissue [27, 28], the necessity of such techniques for the assessment of skeletal muscle remains to be established, as literature findings are inconsistent [29–31]. Moreover, such imaging sequences are rarely used clinically in patients with chronic LBP. On the opposite, T2-weigthed images are routinely obtained when performing lumbosacral MRI examination, have been widely used to assess paraspinal muscle composition in previous studies, and have been shown to provide reliable and accurate calculation of muscle composition when compared to muscle biopsy measurements and spectroscopy [30, 31]. As a result, we believe that the imaging sequence and methodological approach used in this study to quantify muscle composition was adequate.

Although Engstrom et al. previously developed an automated algorithm for the segmentation of the quadratus lumborum muscle [32], the assessment of muscle composition (e.g. threshold) was not addressed by this group. We are aware of only one recent study that has developed a semi-automated interactive tool for the assessment of paraspinal muscle composition, which considerably simplified the task of paraspinal muscle composition assessment [18]. However, threshold values using the interactive tool are based on visual inspection, thus remain rater-dependent. Agreement with a reference method and reliability estimates were not reported. Previous studies examining the reliability of FCSA measurements using a manual thresholding technique, have reported intra-rater ICCs varying between 0.81 and 0.99 [33–35], which were corroborated by our study results. The manual thresholding technique used in the present study has also been found to have excellent inter-software agreement when measurements were obtained with ImageJ and OsiriX [11].

### Study limitations

While accurate and time-efficient, certain difficulties remain with the described automated thresholding algorithm. First, a selected muscle ROI cannot be corrected once it is fully traced. Thus, if the rater is not satisfied with the selected ROI, the segmentation needs to be repeated. We are currently working on the coding of the algorithm to modify this feature, and allow for the correction of the ROI. Second, the algorithm operates on a single slice (jpeg format), thus slice location is important and volume measurements (cm^3^ or mm^3^) cannot be directly obtained. Finally, for accurate measurement of muscle CSA and fat area, the rater needs to indicate the MRI matrix size in the algorithm command prior to performing any measurement.

## Conclusion

In conclusion, we present an automated thresholding algorithm for the assessment and quantification of paraspinal muscle size and composition using axial T2-weighted MR images. The ROI of interest is first manually segmented and then the algorithm computes the muscle total CSA, fat CSA and fat percentage automatically. This novel algorithm was validated against paraspinal muscle composition measurements obtained using an established, highly reliable manual thresholding method, on a sample representing a clinically relevant population with chronic LBP. Our results suggest that the paraspinal muscle composition measurements obtained with the automated algorithm are in excellent agreement with those produced by the manual thresholding technique, with slightly higher intra-rater reliability indices and smaller SEMs. The proposed automated thresholding algorithm greatly simplifies the complexity and tedious aspects of MR imaging assessment of paraspinal muscle composition, and provides a standardized procedure to facilitate replication and comparison among related studies. We have made the algorithm available online at (https://users.encs.concordia.ca/~hrivaz/codes/SemiAutomatic_Thresholding/) for public academic use. The software is accompanied with a video that provides usage instructions. This algorithm can be implemented on the MRI devices to apply the automatic thresholding directly on the scans. While the development of an automated approach for the ROI selection is challenging, due to the variation in the paraspinal muscle morphology between individuals and spinal levels, we are currently working on the development of an atlas-based automated segmentation algorithm.

## Abbreviations

LBP: low back pain
ICC: intra-class correlation coefficient
SEM: standard error of measurement
MRI: magnetic resonance imaging
CT: computed tomography
GCS: goutallier classification scale
CSA: cross-sectional area
FCSA: functional cross-sectional area
ROI: region of interest
